# Soluble Cyanobacterial Carotenoprotein as a Robust Antioxidant Nanocarrier and Delivery Module

**DOI:** 10.3390/antiox9090869

**Published:** 2020-09-15

**Authors:** Eugene G. Maksimov, Alexey V. Zamaraev, Evgenia Yu. Parshina, Yury B. Slonimskiy, Tatiana A. Slastnikova, Alibek A. Abdrakhmanov, Pavel A. Babaev, Svetlana S. Efimova, Olga S. Ostroumova, Alexey V. Stepanov, Ekaterina A. Slutskaya, Anastasia V. Ryabova, Thomas Friedrich, Nikolai N. Sluchanko

**Affiliations:** 1 Department of Biophysics, Faculty of Biology, Lomonosov Moscow State University, 119991 Moscow, Russia; parshinae@gmail.com (E.Y.P.); p.babaew@yandex.ru (P.A.B.); nikolai.sluchanko@mail.ru (N.N.S.); 2 A.N. Bach Institute of Biochemistry, Federal Research Center of Biotechnology of the Russian Academy of Sciences, 119071 Moscow, Russia; santloxan@bk.ru; 3 Faculty of Basic Medicine, MV Lomonosov Moscow State University, 117192 Moscow, Russia; a-zamaraev@yandex.ru (A.V.Z.); alibek.aaa.94@gmail.com (A.A.A.); 4 Center for Strategic Planning and Management of Medical and Biological Health Risks, 119121 Moscow, Russia; 5 Institute of Gene Biology, Russian Academy of Sciences, 119334 Moscow, Russia; slacya@gmail.com; 6 Institute of Cytology of the Russian Academy of Sciences, 194064 St. Petersburg, Russia; ssefimova@mail.ru (S.S.E.); osostroumova@mail.ru (O.S.O.); 7 M.M. Shemyakin and Yu.A. Ovchinnikov Institute of Bioorganic Chemistry, Russian Academy of Sciences, 117997 Moscow, Russia; stepanov.aleksei.v@gmail.com (A.V.S.); slutskay@yandex.ru (E.A.S.); 8 A.M. Prokhorov General Physics Institute, Russian Academy of Sciences, 119991 Moscow, Russia; nastya.ryabova@gmail.com; 9 Institute of Chemistry PC 14, Department of Bioenergetics, Technische Universität Berlin, 10623 Berlin, Germany; friedrich@chem.tu-berlin.de

**Keywords:** carotenoid, protein-protein interactions, carotenoid delivery, protein-membrane interactions, liposomes, oxidative stress

## Abstract

To counteract oxidative stress, antioxidants including carotenoids are highly promising, yet their exploitation is drastically limited by the poor bioavailability and fast photodestruction, whereas current delivery systems are far from being efficient. Here we demonstrate that the recently discovered nanometer-sized water-soluble carotenoprotein from *Anabaena* sp. PCC 7120 (termed AnaCTDH) transiently interacts with liposomes to efficiently extract carotenoids via carotenoid-mediated homodimerization, yielding violet–purple protein samples. We characterize the spectroscopic properties of the obtained pigment–protein complexes and the thermodynamics of liposome–protein carotenoid transfer and demonstrate the delivery of carotenoid echinenone from AnaCTDH into liposomes with an efficiency of up to 70 ± 3%. Most importantly, we show efficient carotenoid delivery to membranes of mammalian cells, which provides protection from reactive oxygen species (ROS). Incubation of neuroblastoma cell line Tet21N in the presence of 1 μM AnaCTDH binding echinenone decreased antimycin A ROS production by 25% (*p* < 0.05). The described carotenoprotein may be considered as part of modular systems for the targeted antioxidant delivery.

## 1. Introduction

Formation of reactive oxygen species (ROS) accompanies electron transfer reactions during aerobic respiration or photosynthesis. Since high ROS levels may be harmful to cells, antioxidants are crucial for maintaining their normal functioning [1,2]. Carotenoids are natural antioxidants playing important roles in photoprotection and regulation of photosynthetic activity of higher plants, algae, and cyanobacteria. Due to the very short lifetime of the excited state [3], carotenoids acting as excitation energy acceptors can rapidly convert light energy into heat, thereby reducing the probability of ROS formation. Mammalian cells cannot produce carotenoids, but some types of the latter are vitally needed not only as antioxidants. For example, β-carotene is a source of retinal, the cofactor of visual photoreceptors [4]. Alongside the reported anti-cancer [5], anti-tumor, or anti-dermatosis abilities of carotenoids [6,7], numerous studies revealed beneficial therapeutic effects of carotenoids in human chronic diseases including the so-called canthaxanthin retinopathy, retinal dystrophy, or aplastic anemia [8]. In any case, from visual pigments to coloration of bird feathers, carotenoids come from diet and must be delivered to specific tissues and cells to perform their functions [9]. While being transported by blood, nutritional carotenoids most often are found in lipoproteins promiscuously accommodating different lipophilic molecules [10]. However, the mechanisms, which allow for the delivery of carotenoids into cells in a specific and targeted manner, are so far unknown.

Modern strategies to deliver carotenoids into tissues are based on liposomes, niosomes, solid lipid nanoparticles, polysaccharides, and oligosaccharides inclusion complexes, which can be produced in a controlled manner [11,12,13,14,15]. The fusion of carotenoid-loaded liposomes with cellular membranes causes delivery of these antioxidants into different cell compartments; however, the efficiency of carotenoid uptake by cells is limited. Reportedly, incubation of cells with liposomes bearing micromolar concentrations of carotenoids embedded in the membrane results in only picomolar concentrations in cells [11]. The efficiency of the delivery is seriously limited by poor carotenoid stability and fast photodestruction. For targeted delivery, a conjugation with antibodies specific to some cell surface components may be necessary, which may interfere with the liposome loading by carotenoids. Alternative protein-based modular constructions are under extensive development, since protein sequence and functionality can be effectively engineered [16,17,18]. Fortunately, natural water-soluble carotenoid-binding proteins may provide the best opportunities for carotenoid transportation and targeted delivery, but this strategy remains completely unexplored. 

The structures of the photoactive orange carotenoid protein (OCP) and some of its recently discovered homologs are optimized by evolution to ensure carotenoid retrieval from membranes [19,20,21], since their physiological function is to deliver the carotenoid molecule to the antenna complexes to quench overexcitation under intense illumination [22]. Besides that, OCP is also an efficient ROS quencher [23]. Upon expression in carotenoid-producing *Escherichia coli* strains, OCP-like proteins can bind different xanthophylls [24,25,26]. Assembly of these water-soluble carotenoproteins requires carotenoid extraction from membranes; however, the mechanism of this process is essentially unknown. Very recently, it was demonstrated in vitro that a natural ~15-kDa homolog of the C-terminal domain of OCP from the cyanobacterium *Anabaena (Nostoc)* sp. PCC 7120 (hereinafter AnaCTDH) can extract ketocarotenoids (echinenone (ECN) and canthaxanthin (CAN)) from membranes of overproducing *E. coli* strains [27,28]. This leads to the maturation of the initially colorless AnaCTDH apoprotein into a violet, soluble nm-sized holoprotein by a process involving carotenoid-mediated protein homodimerization [24,28]. 

To avoid the heterogeneity associated with the uncontrolled lipid, carotenoid, and protein content of *E. coli* membranes used in previous work [27], we employed a simpler model system by selecting artificial liposomes to study the assembly of the water-soluble carotenoid nanocarrier. In contrast to our initial expectations, we found that carotenoid transfer between lipid membranes and the protein is reversible and that the efficiency of this process critically depends on particular protein–membrane and protein–carotenoid interactions. Furthermore, we demonstrate that soluble cyanobacterial carotenoid carriers can be used for the delivery of carotenoids into mammalian cells to decrease the mortality hazard caused by ROS. We discuss the outreach of such approaches for biomedical applications.

## 2. Materials and Methods

### 2.1. Materials 

Synthetic 1,2-diphytanoyl-*sn*-glycero-3-phosphocholine (DPhPC), 1,2-dioleoyl-*sn*-glycero-3-phosphocholine (DOPC), 1,2-dipalmitoyl-*sn*-glycero-3-phosphocholine (DPPC), 1,2-dimyristoyl-*sn*-glycero-3-phosphocholine (DMPC), sphingomyelin (Brain, Porcine) (SM), 1′,3′-bis [1,2-dimyristoyl-*sn*-glycero-3-phospho]-*sn*-glycerol (sodium salt) (TMCL), cholesterol (CHOL) and 1,2-dipalmitoyl-*sn*-glycero-3-phosphoethanolamine-*N*-(lissamine rhodamine B sulfonyl) (Rh-DPPE) were obtained from Avanti Polar Lipids, Inc. (Pelham, AL, USA). Nonactin, NaCl, NaOH, 2-[4-(2-hydroxyethyl)piperazin-1-yl]ethane sulfonic acid (HEPES), 2,2′,2′’,2′’’-(ethane-1,2-diyldinitrilo)tetraacetic acid (EDTA), and hexadecane were purchased from Sigma Chemical (St. Louis, MO, USA).

### 2.2. Cloning, Protein Expression, and Purification

The identity of the constructs and the presence of mutations were verified by DNA sequencing (Evrogen, Moscow, Russia). The obtained plasmids were used to transform chemically competent cells. Proteins were expressed using induction by 1 mM isopropyl-β-thiogalactoside (IPTG) in the presence of kanamycin and ampicillin. 

Apo- and Holoforms of AnaCTDH, and holoforms of COCP (C-terminal domain of OCP from *Synechocystis* sp. PCC 6803 [24,28]), RCP (*Synechocystis* sp. PCC 6803 [20]), wild-type OCP from *Synechocystis* sp. PCC 6803 and the corresponding variant OCP^AA^ (harboring substitutions Y201A/W288A), were expressed in ECN and CAN-producing *Escherichia coli* cells essentially as described earlier [29]. All 6xHis-tagged proteins were purified by immobilized metal-affinity and size-exclusion chromatography to electrophoretic homogeneity and stored at 4 °C in PBS buffer (pH 7.4) supplemented with 3 mM sodium azide. Protein concentrations were determined at 280 nm using calculated protein-specific molar extinction coefficients. 

### 2.3. Lipid Bilayer Setup, Recording System, and Calculations 

Virtually solvent-free planar lipid bilayers were prepared using a monolayer-opposition technique [30] on a 50-µm-diameter aperture in a 10-µm-thick Teflon film separating two (cis- and trans-) compartments of a Teflon chamber. The aperture was pretreated with hexadecane. Lipid bilayers were made from pure DOPC or pure DPhPC. The bath solution contained 0.1 M NaCl, 1 mM EDTA, and was buffered by 5 mM HEPES-NaOH at pH 7.4. After the membrane was completely formed and stabilized, the AnaCTDH apoprotein was added to the cis compartment from a stock solution in storage buffer to obtain final concentrations ranging from 1 to 5 μM. Ag/AgCl electrodes with 1.5% agarose/2 M KCl bridges were used to apply the transmembrane voltage (V) and measure the transmembrane current. ‘Positive voltage’ refers to the case in which the cis side compartment is positive with respect to the trans-side.

Current measurements were carried out using an Axopatch 200B amplifier (AutoMate Scientific Inc., Berkeley, CA, USA) in the voltage clamp mode. Data were digitized by a Digidata 1440A interface and analyzed using pClamp 10 (AutoMate Scientific Inc., Berkeley, CA, USA) and Origin 8.0 (OriginLab Corp., Northampton, MA, USA) software. Data acquisition was performed with a 5 kHz sampling frequency and low-pass filtering at 100 Hz. The current tracks were processed through an eight-pole Bessel 100-kHz filter.

The threshold voltages (*V_bd_*) that cause electrical breakdown of pure DOPC (or pure DPhPC) membranes before and after addition of the AnaCTDH apoprotein into the bath solution up to 1–5 µM, were measured using ramps from 0 to ±500 mV (±10 mV/s). The breakdown voltage was detected from the sharp increase in transmembrane current, which occurs at a certain voltage. No difference between positive and negative voltages was observed.

### 2.4. Measurement of the Membrane Boundary Potential

The steady-state conductance of the planar lipid membranes induced by the alkali metal-specific ionophore nonactin was modulated by one-sided addition of the AnaCTDH apoprotein from a 140 mM stock solution in storage buffer to the membrane-bathing solution (0.1 M NaCl, 1 mM EDTA, 5 mM HEPES-NaOH, pH 7.4) to obtain the threshold concentration (determined by the voltage clamp method) at which the compounds increase the ion permeability of the lipid bilayer. The membranes were composed of DOPC or DPhPC. The conductance of the lipid bilayer was determined by measuring the transmembrane current at a constant transmembrane voltage (V = 50 mV). The subsequent calculations were performed assuming that the membrane conductance *G* is related to the membrane boundary potential difference (*φ_b_*), the potential drop between the aqueous solution and the membrane hydrophobic core, by the sigmoidal Boltzmann-type function [31]

(1) $$\varphi_{b}=\frac{kT}{e}\times \ln\left(\frac{G_{m}}{G_{m}^{0}}\right)$$

here, $G_{m}$ and $G_{m}^{0}$ are the steady-state membrane conductances induced by nonactin in the presence ($G_{m}$) and absence ($G_{m}^{0}$) of protein, respectively, *e* is the elementary charge, *k* the Boltzmann constant and *T* the absolute temperature (in K). The changes in boundary potential (Δ*φ_b_*) for defined experimental conditions were averaged based on at least three independent experiments (mean ± SD).

### 2.5. Confocal Microscopy of Unilamellar Vesicles

Giant unilamellar vesicles were formed by the electroformation method on a pair of indium tin oxide slides using a commercial Vesicle Prep Pro device (Nanion, Munich, Germany) as previously described [32] (standard protocol according to manufacturer’s instructions, 3 V, 10 Hz, 1 h, 55 °C). The lateral phase segregation of membrane components was visualized by introducing a fluorescence-labeled lipid probe (Rh-DPPE) into the source lipid solution mixture in chloroform (2 mM): (1) 50 mol% DOPC and 50 mol% DPPC; (2) 50 mol% DOPC and 50 mol% DMPC; (3) 50 mol% DOPC and 50 mol% SM; (4) 50 mol% DOPC and 50 mol% TMCL; (5) 67 mol% DOPC and 33 mol% CHOL; and (6) 40 mol% DOPC, 40 mol% SM, and 20 mol% CHOL. The Rh-DPPE concentration in the sample was 1 mol%. Rh-DPPE clearly favors the liquid disordered phase (l_d_) and is excluded from the liquid ordered (l_o_) and gel (s_o_) phases [33]. The obtained liposome suspension was divided into aliquots. An aliquot without protein was used as a control. The experimental samples contained lipid to protein ratio of 500:1, 250:1, and 100:1. Vesicles were observed through immersion lenses 60×/1.42 of a confocal microscope (Olympus, Hamburg, Germany). The preparations were studied at 25 °C. Rh-DPPE was excited by 561 nm light from a He-Ne laser. The total number of counted vesicles in a sample typically was 10–15. All experiments were repeated three times and the most typical results are presented.

### 2.6. Differential Scanning Calorimetry

Differential scanning calorimetry experiments were performed using μDSC 7 EVO microcalorimeter (Setaram Inc., Caluire, France). Giant unilamellar vesicles were prepared from pure DMPC or pure DPPC by the electroformation method as described above. The obtained liposome suspensions contained 4 mM lipid and were buffered by 5 mM HEPES-NaOH at pH 7.4. AnaCTDH apoprotein (from concentrated stock solutions in storage buffer) was added by aliquots, up to the lipid to protein molar ratio of 500:1, 250:1, 100:1, and 70:1. Liposome suspensions were incubated with protein for 30 min at room temperature and then heated at a constant rate of 0.2 K·min^−1^. The reversibility of the thermal transition was assessed by re-heating the sample immediately after the cooling step from the previous scan. At least three independent experiments were performed for each system/protein. The temperature dependence of the excess heat capacity was analyzed using the Calisto Processing software (Setaram Instrumentation, Caluire, France). The thermal behavior of the liposome suspension in the absence and presence of the protein was described by the changes in the temperature of the maximum of the excess heat capacity, T_m_ (indicative of the main phase transition), and the width at half-maximum of the main peak, T_1/2_ (corresponding to the cooperativity of the lipid phase transition)_,_ of the heat capacity curve.

### 2.7. Thin Layer Chromatography of Carotenoids

Carotenoids were extracted from membranes of ECN/CAN-producing *E. coli* strains after two days of carotenoid synthesis [24,29] or from carotenoproteins by the addition of equal volume of pure acetone, followed by the addition of equal volume of kerosene. Aliquots of the colored carotenoid-enriched fraction clarified by centrifugation were subjected to thin-layer chromatography on silica gel plates (type Silufol, Kavalier, Prague, Czechoslovakia). Thin layer chromatography was run in a closed glass chamber using a mixture of kerosene (80% *v*/*v*) and acetone (20% *v*/*v*) during 15 min at room temperature. The TLC plates were photographed immediately to avoid oxidation and photodamage of carotenoids. Previous work reporting R_f_ values for different carotenoids was used as a reference [34] for identifying the carotenoids on TLC plates.

### 2.8. Production of Liposomes with Carotenoids

CAN and ECN were extracted from aqueous solutions of COCP holoprotein (the C-terminal domain of OCP [34]) and wild-type OCP, respectively, by chloroform. For this, a three-fold volume of chloroform was added to a protein solution, vigorously stirred and incubated overnight at +37 °C. After the incubation, the resulting mixture was centrifuged at 12,000 rpm for 15 min, and the carotenoid solution in chloroform was carefully removed from the lower part of the test tube. The precipitate was washed with chloroform once again. The resulting carotenoid solution in chloroform (5 mL) was evaporated in a rotary evaporator to a volume of 1 mL. Liposomes with carotenoids were obtained according to [35] with minor modifications. 100 μL of chloroform solution (20 mg/mL) of egg phosphatidylcholine (Avanti) was added to the carotenoid solution in chloroform (1 mL), stirred, and chloroform was evaporated in a rotary evaporator. The resulting lipid film with carotenoids was solubilized in 500 μL sodium-phosphate buffer (10 mM sodium phosphate, 150 mM sodium chloride, pH 8.0) with subsequent sonication (model Finnsonik W-181-T, FinnSonic Oy, Lahti, Finland) at a frequency of 40 kHz and a power of 90 watts for 30 min. Then, the resulting suspension of liposomes was centrifuged at 6000 rpm for 5 min for purification from aggregates and carotenoids not incorporated into the liposomes. The supernatant, containing purified liposomes was filtered four times through a filter with an average pore diameter of 0.2 μm (Merck Millipore, Burlington, MA, USA) for standardizing the size of liposomes. The filter was washed with another 100 μL of sodium phosphate buffer (pH 7.4) and combined with filtered suspension of liposomes. The resulting liposomes were stored in the dark at +4 °C under argon atmosphere. The typical size of the liposomes was about 200 ± 100 nm, which was tested by dynamic light scattering (Zetasizer Nano ZS, Malvern Panalytical Ltd., Malvern, UK).

### 2.9. Absorption Measurements

Absorption spectra were recorded using a MayaPro2000 spectrophotometer (Ocean Optics, Dunedin, FL, USA). In order to compensate for the effect of light scattering, an integrating sphere BIM-3003 (Hangzhou Brolight Technology Ltd., Hangzhou, China) was installed in front of the sample. The kinetics of carotenoid transfer were measured as the change of optical density at 550 nm with 100 ms time resolution, the precision of the optical density measurement was 5 × 10^−3^ OD units. The temperature of the samples was stabilized by a Peltier-controlled cuvette holder Qpod 2e (Quantum Northwest Inc., Liberty Lake, WA, USA) with a magnetic stirrer. To estimate the efficiency of the carotenoid transfer under the given conditions, spectral decomposition using reference spectra of carotenoid donors and carotenoid acceptors in 100% holoform was performed using OriginPro 9.0 software (OriginLab Corp., Northampton, MA, USA) by fitting the corresponding contributions of the donor and acceptor spectrum to the spectrum obtained at the end of the mixing experiment. All experiments were performed three times and the most typical results are presented.

### 2.10. Raman Spectroscopy Measurements

Resonance Raman spectra of carotenoids in proteins or in liposomes were obtained under continuous excitation at 532 nm. The laser beam was focused on a 0.1 mm glass capillary containing the sample. Raman-scattered light was collected and subsequently imaged using a confocal microscope-based system (model Ntegra Spectra, NT-MDT Spectrum Instruments, Zelenograd, Russia). The same system was used for Raman spectroscopy and imaging of HeLa cells enriched by carotenoids. Processing of Raman images was performed using Nova (NT-MDT Spectrum Instruments, Zelenograd, Russia) and ImageJ software (Available online: https://imagej.net/Downloads, accessed on 10 September 2020). At least 10 different HeLa cells were analyzed. A characteristic overlay of Raman signature intensity (ν_1_ band at 1522 cm^−1^ minus background at 1550 cm^−1^) with the image of HeLa cell in transmitted light is presented.

### 2.11. Delivery of Carotenoids into Mammalian Cells by AnaCTDH

HEK293T (human embryonic kidney epithelial cells, ATCC^®^ CRL-3216), HeLa (human cervical cancer epithelial cells), and TET21N (human neuroblastoma cells) were obtained from ATCC and as a gift from Division of Toxicology, Karolinska Institute (Stockholm, Sweden). The HeLa cell line was cultured in complete DMEM medium (ThermoFisher Scientific, Waltham, MA, USA) with 10% (*v*/*v*) heat-inactivated fetal calf serum (ThermoFisher Scientific), 100 U/mL penicillin/streptomycin (PanEco, Moscow, Russia) in a humidified atmosphere with 5% CO_2_ at 37 °C. For confocal Raman microscopy, HeLa cells were cultured on glass bottom dishes (POC-R2 Cell Cultivation System, PeCon GmbH, Germany) overnight in 5% CO_2_ at 37 °C. The cells were incubated in a growth medium, containing 1 µM AnaCTDH holoprotein for 2 h at 37 °C.

The distribution of the TagRFP-AnaCTDH chimera after incubation of cells was recorded using an LSM-710 laser scanning confocal microscope (Carl Zeiss Microscopy, Jena, Germany). Fluorescence was exited at 561 nm, the emission was detected between 565 and 730 nm. The 63× oil immersion Plan-Apochromat objective with numerical aperture of 1.4 (Carl Zeiss Microscopy, Jena, Germany) was used to obtain high-quality images.

Fluorescence lifetime images were recorded using a FLIM module connected to the LSM-710-NLO (Becker & Hickl GmbH, Berlin, Germany), consisting of a time-correlated photon counting system (TCSPC) SPC-150, hybrid photodetector GaAsP HPM-100-07, and SPCImage software. The TagRFP-AnaCTDH chimera was two-photon exited with 1050 nm provided by a femtosecond laser (model Cameleon Ultra II, Coherent Inc., Santa Clara, CA, USA).

### 2.12. Antioxidant Activity of Carotenoids Delivered into Mammalian Cells by CTDH

Assessment of ROS production was performed using dihydroethidium (DHE) (Sigma-Aldrich, St. Louis, MO, USA) and 2′,7′-Dichlorofluorescin diacetate (DCFDA) (Sigma-Aldrich, St. Louis, MO, USA), which are indicators of hydroxyl, peroxyl, and superoxide radicals, and other reactive oxygen species (ROS) within the cell, according to the manufacturer’s protocols. Experiments were carried out using FACS Canto II (Becton Dickinson, Franklin Lakes, NJ, USA) flow cytometer.

To analyze the antioxidant effect of carotenoids we used the neuroblastoma cell line Tet21N and employed antimycin A (AMA) as an inhibitor of electron transport in mitochondria, which has been used as a ROS generator in biological systems. AMA inhibits succinate oxidase and NADH oxidase, and also inhibits mitochondrial electron transport between cytochrome b and c [36]. This inhibition causes the collapse of the proton gradient across the mitochondrial inner membrane and production of ROS.

Each experiment was conducted at least three times unless another number is stated in the captions of the corresponding figures.

### 2.13. Statistical Analysis

Student’s *t*-test and analysis of variance (ANOVA) were performed to compare the mean of the control group to the mean of the treatment group. *p* < 0.05 was considered significant. Normality of distribution was examined by using Shapiro–Wilk normality test. The data were considered as normally distributed if *p* > 0.05. Equality of variance was examined with an F test. *p* > 0.05 was considered as equal variances. The data shown are mean values of three independent experiments with error bars corresponding to standard errors. All statistical analysis was performed by using OriginPro 2015 (OriginLab Corp., Northampton, MA, USA).

## 3. Results and Discussion

### 3.1. Direct Interaction of AnaCTDH Apoprotein with the Membranes

**Figure 1:**
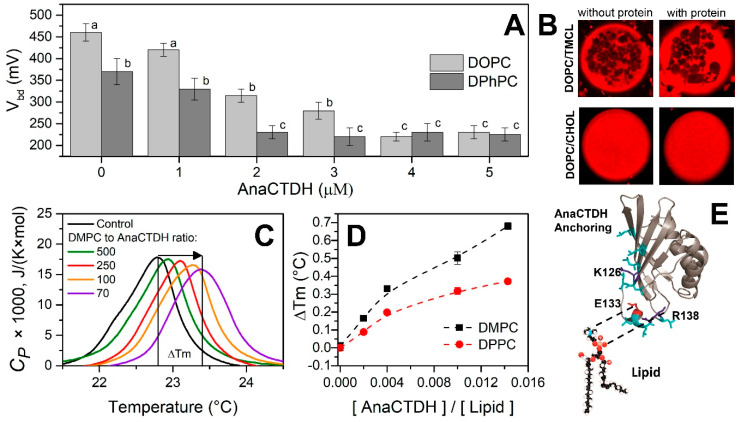
Interactions of the AnaCTDH apoprotein with membranes. (**A**) Threshold voltages (*V_bd_*) that caused electrical breakdown of DOPC and DPhPC membranes at different concentrations of the AnaCTDH apoprotein in solution. All data are reported as mean values ± SD from three independent experiments. The means accompanied by different letters (a, b, and c) are significantly different at *p* < 0.05. (**B**) Fluorescence micrographs of giant unilamellar vesicle membranes made from 50 mol% DOPC and 50 mol% TMCL (top row) and 67 mol% DOPC and 33 mol% CHOL (bottom row) (stained with fluorescent lipid marker) in the absence and in the presence of AnaCTDH apoprotein. The lipid:protein ratio was 100:1. The liquid, disordered (l_d_) phase appears red, while the solid, ordered (s_o_) phase remains dark. Image size is 15 × 15 μm. (**C**) DSC thermograms of DMPC liposomes in the absence (control) and in the presence of the AnaCTDH-apoprotein in a lipid to protein molar ratios indicated using color coding. (**D**) The increase of phase transition temperature of liposomes composed of DMPC and DPPC in response to the increasing AnaCTDH concentration. The results were averaged based on three independent experiments (mean ± SEM). (**E**) Model of the interaction of the AnaCTDH apoprotein with PC. Glu-133 is shown in red (numbering from 6FEJ structure), positively charged residues are shown in blue, leucines are shown in cyan. The structure of the AnaCTDH apoprotein was drawn in Pymol using PDB ID 6FEJ chain A [28].

Upon bacterial expression, the water-soluble AnaCTDH apoprotein is able to mature efficiently on its own by extracting carotenoids from membranes [24,27]. This suggests its direct interaction with the lipid bilayer, prompting us to test this ability using several model membranes (Figure 1). 

The threshold voltages (*V_bd_*) that causes the electrical breakdown of the DPhPC and DOPC membranes in the absence of the AnaCTDH apoprotein were 460 ± 40 mV and 370 ± 20 mV (significantly different, *p* < 0.05), respectively (Figure 1A). The addition of AnaCTDH apoprotein into the membrane bathing solution up to 5 μM led to about two-fold decrease in *V_bd_* of DPhPC-bilayers (230 ± 15 mV, *p* < 0.05). For DOPC-bilayers, *V_bd_* decreased by 1.5 times (down to 225 ± 15 mV, *p* < 0.05). These observations indicate that the AnaCTDH apoprotein interacts with model membranes and thereby decreases their electrical stability of model membranes. In contrast, the addition of the AnaCTDH apoprotein did not affect the steady-state conductance of DPhPC- and DOPC-bilayers induced by the alkali metal ionophore nonactin. This indicates that the distribution of the electrical potential on the membrane/water interface (i.e., membrane boundary potential, φ_b_) remains unchanged upon protein binding (Δφ_b_ = 1 ± 1 mV). Thus, interactions of the AnaCTDH apoprotein and membranes are transient but detectable, which is reasonable considering the putative functioning of this protein as a carotenoid carrier confined in the water phase.

The lateral heterogeneity of vesicle membranes formed from DOPC/DPPC, DOPC/DMPC, DOPC/SM, and DOPC/TMCL mixtures (each 50/50 mol%) before and after addition of AnaCTDH apoprotein into liposome suspensions was studied by imaging the distribution of a fluorescence-labeled lipid probe, which distributes into the liquid disordered (l_d_) phase, by confocal microscopy. In these lipid systems, we observed the heterogeneity related to coexistence of the liquid-disordered (l_d_) and gel-lipid (s_o_) phases (Figure 1B, top row). Micrographs (Figure 1D) demonstrate that addition of the AnaCTDH apoprotein at a lipid:protein molar ratio as high as 100:1 did not change the shape of the lipid vesicles and the phase segregation scenario. 

Although visible phase segregation was not affected by interactions of the AnaCTDH apoprotein with membranes, an appreciable increase of the lipid phase transition temperature was found (Figure 1C). For liposomes composed of DMPC, the phase transition occurs at 22.70 °C. Addition of protein up to a lipid:protein ratio of 70:1 shifts the phase transition temperature by ~0.70 °C (Figure 1C), indicative of the protein–lipid interaction. The width at half-height of the main peak in the excess heat capacity thermogram (T_1/2_), informing about the cooperativity of the phase transition, was not changed. Similar results were obtained with DPPC (Figure 1D), suggesting that the interaction of the protein with the membrane does not depend on the thickness of its hydrocarbon core. This suggests that the AnaCTDH apoprotein likely interacts with the polar heads of neighboring membrane lipids in the model membranes owing to electrostatic interactions. It is likely that the structurally polymorphous C-terminal tail (CTT) of the AnaCTDH apoprotein [28,37], featuring Glu-133 and Arg-138 residues (numbering from 6FEJ structure), can act as an anchor to increase the probability of carotenoid uptake (Figure 1E), whereas several Leu residues may contribute to this process by facilitating carotenoid binding. This is indirectly supported by the fact that in the absence of the CTT, the rate constant of holoprotein formation is reduced [28]. Considering also that, in the crystallographic AnaCTDH apoprotein dimer, the CTT partially blocks the so-called carotenoid tunnel and adopts different conformations, it is likely that this structurally mobile Leu-rich motif plays a critical role in carotenoid transfer processes.

### 3.2. Carotenoid Uptake by the AnaCTDH Apoprotein from Liposomes

It was previously shown that AnaCTDH can efficiently extract ECN and CAN from membranes upon expression in the specific carotenoid-producing *E. coli* strains [24,27]. Moreover, due to the different stability and spectral properties, ECN- and CAN-bound AnaCTDH forms can be readily separated from the resulting mixture [24]. Figure A2 in Appendix A shows a thin layer chromatogram indicating the enrichment of the corresponding carotenoids in distinct fractions of AnaCTDH. 

**Figure 2:**
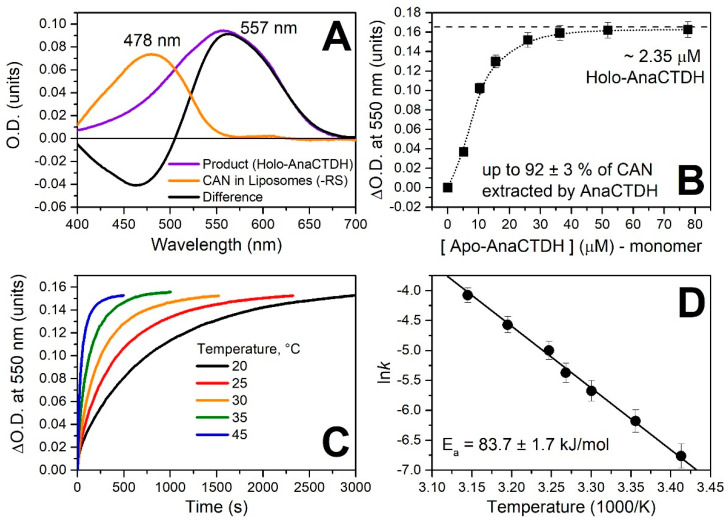
CAN uptake by the AnaCTDH apoprotein from liposomes in PBS (pH 7.4). (**A**) Absorption spectra of CAN in liposomes and after incorporation into AnaCTDH. Rayleigh scattering (RS) was subtracted. (**B**) Increase of O.D. at 550 nm upon addition of different amounts of the AnaCTDH apoprotein to CAN-containing liposomes. (**C**) Time-courses of AnaCTDH holoprotein accumulation due to ECN uptake from liposomes at different temperatures. (**D**) The corresponding temperature dependency of rate constants (Arrhenius plot). All values are presented as mean values ± SD from three independent experiments.

**Figure A1:**
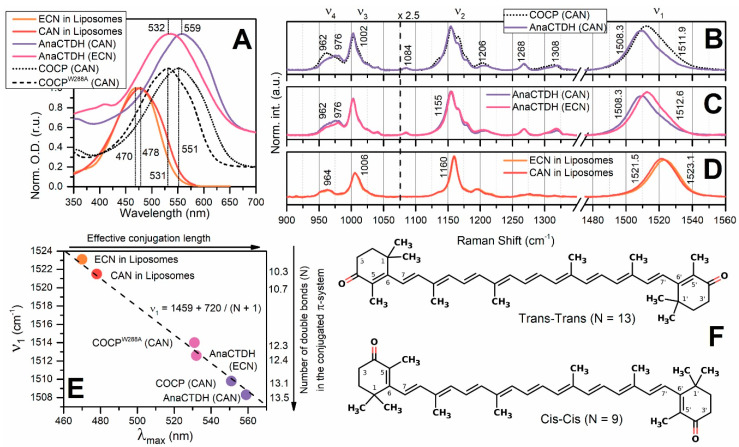
Spectral properties of ECN and CAN in different environments. (**A**) Normalized absorption of AnaCTDH containing CAN (violet) and ECN (purple). Orange and red lines show the absorption of ECN and CAN in liposomes, respectively. Dotted and dashed lines show the absorption of COCP and the W288A sequence variant thereof, respectively, both containing CAN. Spectra were shifted along the *y*-axis for the better presentation. (**B**,**C**) Resonance Raman spectra of CTD-like carotenoid proteins recorded with 532 nm excitation. (**B**) AnaCTDH (solid violet line) and COCP (dotted line), both containing CAN. (**C**) AnaCTDH containing CAN (violet) and ECN (purple). (**D**) Raman spectra of ECN in liposomes. The Raman signal in the 900–1060 cm^−1^ region was magnified 2.5 times for better presentation. Note the *x*-axis break from 1350 to 1475 cm^−1^. (**E**) Dependency of the Raman shift of the -C=C- stretching mode (ν_1_) on the position of the S_0_–S_2_ maximum (λ_max_) in absorption spectra of ECN and CAN embedded in liposomes and proteins. Arrows indicate, how the effective conjugation length and the average number of double bonds changes among samples. The average number of conjugated double bonds was calculated using the empirical dependency denoted in the text [48]. (**F**) Chemical structures of *cis-cis* and *trans-trans* isomers of CAN.

**Figure A2:**
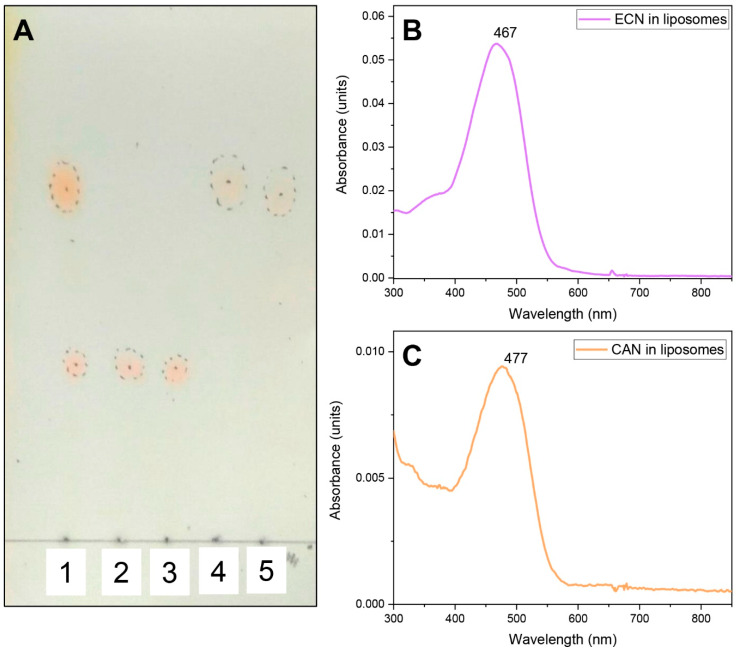
Analysis of ECN and CAN by thin layer chromatography and absorbance spectroscopy. (**A**) thin layer chromatogram in a 80:20 kerosene:acetone (*v*/*v*) system showing the carotenoid content in the membranes of the ECN/CAN-producing *E. coli* strain [29,34] (1) and the samples containing AnaCTDH(CAN) (2), CAN-loaded liposomes (3), AnaCTDH(ECN) (4), or ECN-loaded liposomes (5) used in the study. (**B**) absorbance spectrum of ECN in liposomes obtained by running SEC with full absorbance spectrum detection. The spectrum was corrected for the high scattering from liposomes by subtracting the scattering profile measured on empty liposomes. (**C**) Absorbance spectrum of CAN in liposomes obtained by running SEC with full absorbance spectrum detection. The spectrum was corrected for the high scattering from liposomes by subtracting the scattering profile measured on empty liposomes. Note the difference of the peak maximum position for ECN and CAN.

Being able to obtain pure ECN- and CAN-associated protein fractions and ECN- or CAN-enriched artificial liposomes, we assessed the optical response of these carotenoids in different environments (Figure A1 in Appendix A), which provided us with the spectroscopic signatures to study interactions of liposomes loaded with carotenoids and the AnaCTDH apoprotein (Figure 2). 

**Figure A3:**
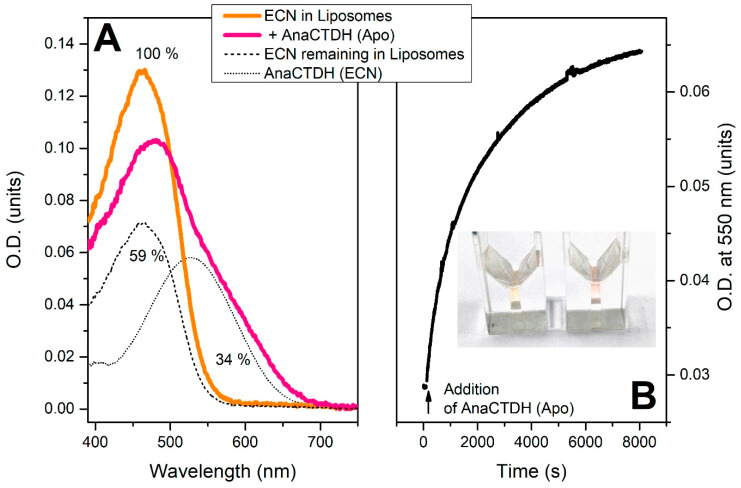
ECN uptake from liposomes by AnaCTDH apoprotein. (**A**) Absorption spectra of ECN in liposomes before (orange) and after (pink) four hours of incubation with 300 µM of AnaCTDH at 30 °C. Resulting spectrum (pink) was decomposed into components which correspond to absorption of ECN remaining in liposomes (dashed line) and in AnaCTDH (dotted line). Percentiles indicate the ECN content in different fractions comparing to the indicial concentration in liposomes. Some fraction of carotenoid (~7%) was lost due to photodegradation upon the continuous absorption measurements. (**B**) Time-course of accumulation of AnaCTDH(ECN) due to carotenoid uptake from liposomes monitored as increase of optical density at 550 nm. The inset shows cuvettes with solutions of ECN-containing liposomes before (left) and after incubation with AnaCTDH apoprotein.

After addition of colorless AnaCTDH apoprotein to yellowish liposomes containing CAN, the solution turned violet, with the concomitant pronounced spectral changes (Figure 2A) characteristic for transfer of the carotenoid into the protein accompanied by homodimerization of the latter [24]. This indicated that AnaCTDH apoprotein interacts also with artificial membranes to efficiently extract the embedded carotenoid molecules. The rate constant of this process depended on temperature (with an activation energy E_a_ ~ 83.7 ± 1.7 kJ/mol) (Figure 2C,D). Titration experiments monitoring the increase of the absorption at 550 nm yielded saturation of the effect at 10 AnaCTDH apoprotein monomers per single carotenoid molecule (Figure 2B). This indicates a moderate efficiency for carotenoid uptake and is in good agreement with the idea of an unstable, transient AnaCTDH apoprotein interaction with the membranes shown above. At an excess of the AnaCTDH apoprotein, almost all CAN molecules were extracted from liposomes, with up to 92 ± 3% inserted into the violet AnaCTDH (CAN) holoprotein. In contrast, only ~34 ± 4% of ECN was extracted by the AnaCTDH apoprotein from ECN-containing liposomes (Figure A3 in Appendix A). Accumulation of the AnaCTDH(ECN) holoprotein was almost six times slower compared to the AnaCTDH(CAN) holoprotein at 30 °C and similar protein concentrations.

### 3.3. Carotenoid Delivery into Liposomes

**Figure 3:**
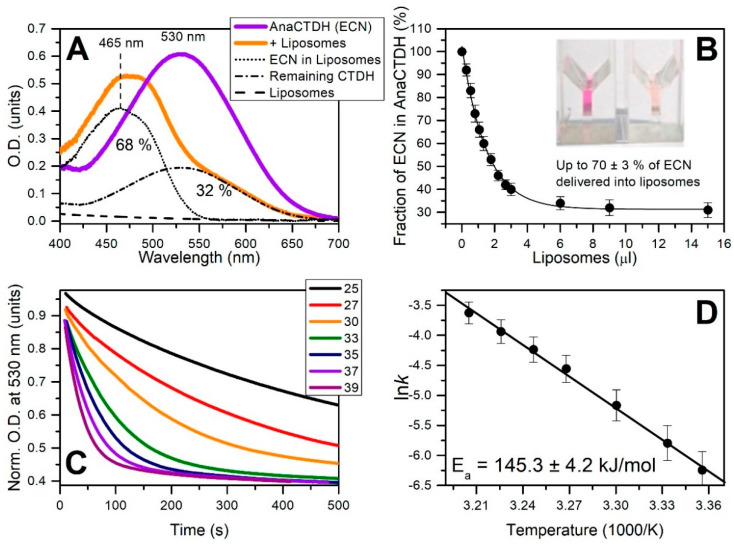
ECN delivery by AnaCTDH(ECN) holoprotein into liposomes. (**A**) The absorption spectrum of AnaCTDH(ECN) before (purple) and after addition of liposomes (orange). The percentage of carotenoid remaining in AnaCTDH after addition of liposomes was determined by decomposing the resulting spectrum into AnaCTDH(ECN) absorption and scattering of liposomes, considering also the fact that ECN absorption in liposomes, peaked at ~465 nm, is significantly blue-shifted and does not overlap with protein-bound ECN in the 550–700 nm region. (**B**) The percentage of carotenoid remaining in AnaCTDH upon increasing the concentration of liposomes in solution. Values are presented as mean ± SD from three independent experiments. The dependency was approximated by an exponential function in order to determine the maximum yield of carotenoid delivery. The inset shows cuvettes with the AnaCTDH(ECN) holoprotein solution before (left) and after (right) incubation with liposomes. (**C**) Time-courses of optical density at 530 nm corresponding to the disappearance of the AnaCTDH(ECN) holoprotein due to carotenoid delivery into liposomes at different temperatures (shown in °C). (**D**) The corresponding temperature dependence of rate constants (Arrhenius plot). All values are presented as mean ± SD from three independent experiments.

Since ~65 ± 4% ECN remains in liposomes even with the AnaCTDH apoprotein present in excess, one can expect this to reflect an equilibrium between carotenoid uptake from and carotenoid delivery into membranes. In theory, the equilibrium can be shifted to make the delivery more efficient, which prompted us to test different carotenoprotein holoforms as carotenoid donors for the liposomes. Upon mixing of CAN-containing COCP (C-terminal domain of *Synechocystis* OCP) [38], OCP^AA^ [39], RCP [20], and AnaCTDH holoproteins with liposomes, we failed to observe any substantial carotenoid delivery. In contrast, the addition of AnaCTDH(ECN) holoprotein led to a decrease of 530 nm absorption and a concomitant increase of 460 nm absorption (Figure 3A). This could be followed visually as a color change from violet-purple to light yellow, indicating productive carotenoid delivery from the AnaCTDH(ECN) holoprotein to liposomes. Upon titration by liposomes, saturation of ECN transfer was reached corresponding to a maximum carotenoid delivery of ~70 ± 3% (Figure 3B).

**Figure 4:**
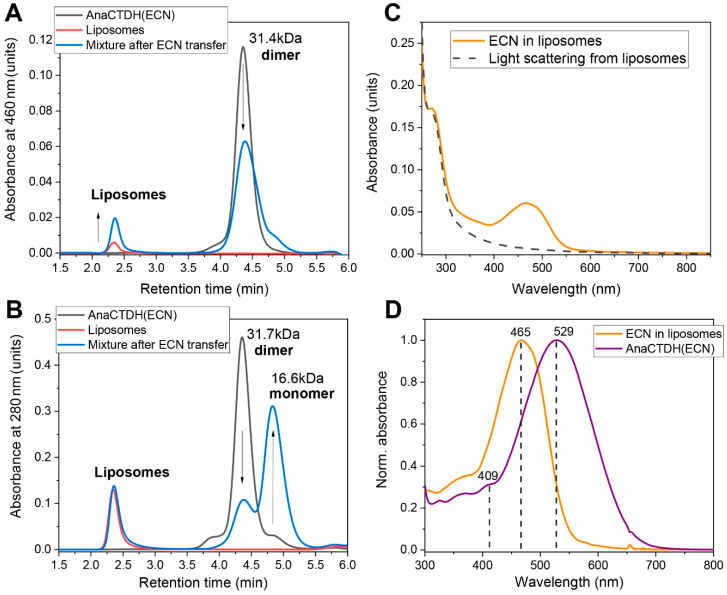
Carotenoid delivery from AnaCTDH(ECN) holoprotein to liposomes studied by size-exclusion spectrochromatography. (**A**,**B**) Elution profiles of AnaCTDH(ECN), liposomes, or their mixture pre-incubated for 40 min at 30 °C to ensure carotenoid transfer, followed by either 460 nm (**A**) or 280 nm (**B**) absorbance, using a Superdex 200 Increase 5/150 column (GE Healthcare) operated at a 0.45 mL/min flow rate by a Varian ProStar 335 diode-array system with the full spectrum absorbance detection at a 0.45 mL/min flow rate. Apparent Mw for the protein peaks was estimated from column calibration using protein standards (indicated in kDa). Note that liposomes elute in the void volume and give significant light scattering. Arrows indicate the observed changes as the result of carotenoid transfer from AnaCTDH (ECN) to liposomes accompanied by AnaCTDH dissociation into apoprotein monomers. Note that protein does not migrate to the liposome fraction, indicating the absence of tight binding. The most typical result is shown. (**C**) Retrieving the ECN absorption spectrum taking into account light scattering from sample containing pure liposomes in the absence of ECN. (**D**) Normalized absorbance spectra of the initial AnaCTDH(ECN) preparation and ECN in liposomes at the end of the transfer (normalization to the peak absorbance as indicated).

The rate constant of ECN delivery into liposomes by AnaCTDH(ECN) was very sensitive to temperature, yielding an E_a_ value of ~145.3 ± 4.2 kJ/mol (Figure 3C,D), which is larger than the activation energy barrier for CAN uptake by AnaCTDH apoprotein and suggests significant rearrangements of the AnaCTDH protein conformation upon carotenoid release. It is worth noting that the ECN delivery rate constant is significantly higher than the rate constant of ECN uptake at 30 °C (Figure A3 in Appendix A) and could be even faster at 37 °C, providing a solid thermodynamic foundation for ECN delivery at physiological temperatures. The physical translocation of ECN from the AnaCTDH(ECN) holoprotein into the liposome membranes accompanied by the formation of the empty AnaCTDH apoprotein monomer were directly confirmed by size-exclusion spectrochromatography (Figure 4).

### 3.4. Carotenoid Delivery from AnaCTDH(ECN) Holoprotein into Mammalian Cells

**Figure 5:**
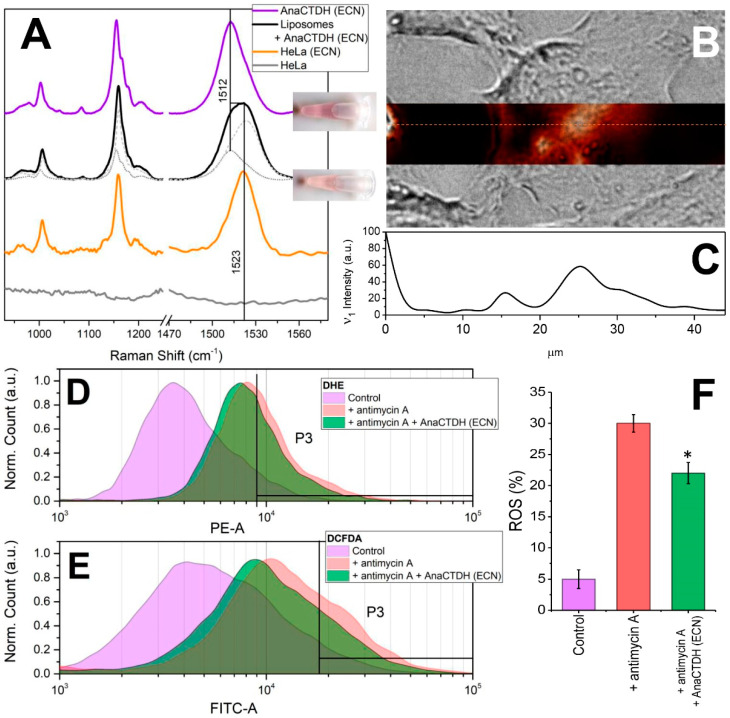
Carotenoid delivery by AnaCTDH(ECN) to mammalian cells. (**A**) Raman spectra of ECN in AnaCTDH (ECN) (violet) and in liposome suspensions after incubation with AnaCTDH (ECN) (black). The spectrum was decomposed into a linear combination of Raman spectra of ECN in AnaCTDH (ECN) (30%) and in liposomes (70%) (see Figure A1). The orange line shows characteristic Raman spectra of ECN in HeLa cells. The grey line shows Raman spectrum of HeLa cells before incubation with AnaCTDH(ECN), with no bands in the carotenoid region observed. The inset shows a tube with a suspension of HeLa cells containing 5 μM of AnaCTDH(ECN) before (top) and after two hours of incubation (bottom) at 37 °C. (**B**) Overlay of microscopic images of a typical HeLa cell in transmitted light with the of ν_1_ band intensity (at 1522 cm^−1^ minus background at 1550 cm^−1^) presented in pseudo colors. The dotted line shows a cross section through the Raman image, the corresponding distribution of the ν_1_ band signature (intensity at 1522 cm^−1^ minus background at 1550 cm^−1^) is shown in (**C**). Effect of carotenoid delivery by AnaCTDH(ECN) into Tet21N cells on ROS production induced by antimycin A was determined by the fluorescence of the dyes DHE (**D**) and DCFDA (**E**) followed by flow cytometry. (**F**) The yield of ROS in Tet21N cells (purple, control sample), under oxidative stress induced by antimycin A treatment (red) and in cells pre-incubated with CTDH (ECN) and treated by antimycin A (green). Values are presented as mean and standard deviation. * indicates significance at the level of *p* < 0.05 compared with cells exposed to antimycin A only (statistical analysis performed with the t-Student’s test at significance level of 5%).

We next questioned whether AnaCTDH-mediated ECN delivery can occur with more complex, biologically relevant membrane models. After incubation of HEK293, HeLa, neuroblastoma (Tet21N), and ovary carcinoma cell suspensions in the presence of AnaCTDH(ECN) holoprotein, characteristic color changes of the protein-containing suspension from purple-violet into yellow (Figure 5) were observed, pointing to ECN delivery into the cell membranes similar to the process observed with liposomes (see Figure 3).

**Figure A4:**
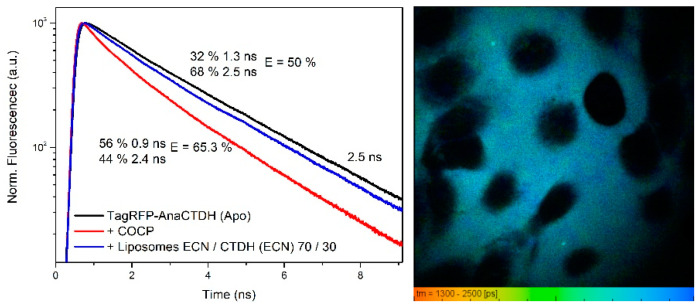
**Left**: fluorescence decay kinetics of the TagRFP-AnaCTDH chimera in the absence of carotenoid (black), after transfer of CAN from COCP (red) and after addition of liposomes (blue) which were previously enriched by ECN via the AnaCTDH(ECN) holoprotein. **Right**: FLIM image of HeLa cells with the TagRFP-AnaCTDH chimera showing no signs of colocalization of the protein at the membranes.

Measurements of ECN absorption in eukaryotic cell lines are complicated due to significant light scattering. To circumvent this difficulty, characteristic Raman signatures (see Figure A1 and description in Appendix A) were used to study carotenoid delivery and distribution in cells. Figure 5A shows that the Raman spectrum of AnaCTDH(ECN) changes after incubation with liposomes: the ν_1_ band becomes significantly broader due to the contributions from two different fractions of ECN: one embedded in AnaCTDH (~30%), while the other fraction resides in membranes. The same distribution was observed upon incubation of HeLa cells in the presence of AnaCTDH(ECN) (data not shown). After washing out the residual AnaCTDH protein with fresh culture medium, we were able to analyze intracellular carotenoid distribution by Raman microscopy. After incubation with AnaCTDH(ECN), the normally carotenoid-free HeLa cells demonstrated characteristic spectral signatures of ECN in membranes, while the contribution from AnaCTDH(ECN) completely vanished. Using the microscope, we found that the Raman signatures of ECN colocalized with the cells (Figure 5B). Notably, the carotenoid distribution across the cell, which could be estimated by the intensity of ν_1_ band, was not homogeneous (Figure 5C). Importantly, a fusion protein between a red fluorescent protein (TagRFP, N-terminal), which facilitates fluorescence imaging, and AnaCTDH (C-terminal) did not prevent ECN extraction from different sources (proteins or liposomes) by the AnaCTDH moiety (Figure A4 in Appendix A) despite the larger size of TagRFP (26 kDa) compared to AnaCTDH (15 kDa). This strongly supports the applicability of the proposed AnaCTDH carotenoid nanocarrier as part of prospective modular systems for targeted carotenoid delivery, in which the desired targeting modules can be attached to the N-terminus of AnaCTDH. Moreover, according to FLIM data, the TagRFP-AnaCTDH chimeric protein did not localize within the cells (Figure A4 in Appendix A), which excludes protein adsorption or endocytosis as the reason for the observed increased carotenoid content of cells detected by Raman signatures. Based on these observations, we conclude that AnaCTDH(ECN) approaches the outer cell membrane, unloads the carotenoid, and remains outside the cell, while carotenoid is likely redistributed across cellular membranes due to other, internal transport mechanisms.

### 3.5. Carotenoid Delivery to Mammalian Cells Alleviates Oxidative Stress

Having found that ECN could be delivered to the plasmalemma of cells, we tested if this natural antioxidant can counteract ROS production from intracellular (mitochondrial) sources. Using dihydroethidium (DHE) and 2′–7′-dichlorodihydrofluorescein diacetate (DCFDA) staining protocols we analyzed ROS accumulation by flow cytometry. The addition of antimycin A [36] to the Tet21N cell line induces ROS production from 5% up to 30% (*p* < 0.01). As a positive antioxidant control, we used *N*-acetylcysteine (NAC) which has a free radical-scavenging property and almost completely prevents the accumulation of ROS after the antimycin A treatment in Tet21N cells [40]. Incubation of cells in the presence of 1 μM AnaCTDH(ECN) decreased ROS production by 25% (from 30% to 22%, *p* < 0.05) in both types of experiments (DHE and DCFDA staining) (Figure 5D–F). Thus, ECN delivered into mammalian cells by the cyanobacterial protein can protect them from oxidative stress.

## 4. Conclusions

**Figure 6:**
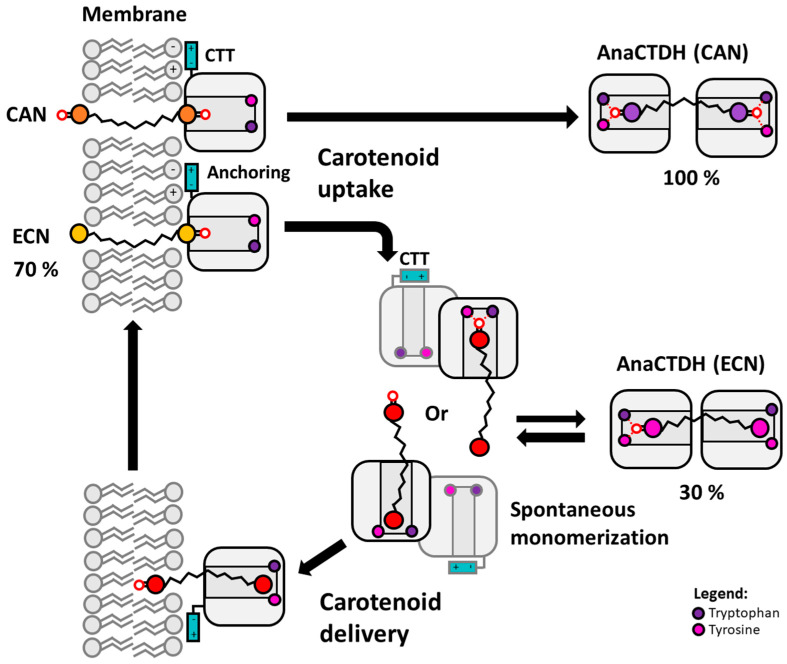
Proposed model for the AnaCTDH-mediated carotenoid uptake and delivery. Carotenoid uptake by AnaCTDH from the membrane is promoted by electrostatic interactions of the CTT and lipid head groups resulting in anchoring and formation of a transient complex between the membrane and the protein facing its carotenoid binding cavity towards the membrane. In such a complex, spontaneous translocation of the carotenoid into the hydrophobic part of the protein may be stabilized by the formation of the hydrogen bonds between the carotenoid keto group and the conserved Trp/Tyr residues of AnaCTDH. Due to a significant length of the carotenoid molecule, it requires two AnaCTDH subunits to isolate it from the solvent. The presence of two keto groups in CAN results in most efficient carotenoid binding in the AnaCTDH dimer, while ECN binding is apparently weaker. Since both types of AnaCTDH holoproteins can transfer carotenoids into other proteins, we postulate that intermediary, spontaneous monomerization of the protein dimer occurs regardless of the carotenoid type [24]. However, only AnaCTDH monomers in which keto group of ECN loses connection with the protein give the carotenoid an opportunity to escape another protein subunit and return to the membrane.

We found that AnaCTDH transiently interacts with the membrane, which appears to be a critical step for the formation of water-soluble carotenoid holoproteins. Comparing the optical response of CTDH with different embedded ketocarotenoids (ECN or CAN) we have found that the stability of protein-carotenoid complex strongly depends on the presence of hydrogen bonds between the keto group of the carotenoid and the conserved aromatic residues. This may be an interesting subject for engineering to modulate the carotenoid binding selectivity and efficiency in future research endeavors. As demonstrated by comparison of the CAN- and ECN-bound forms of AnaCTDH, the relative stability of protein–chromophore interactions determines the ability of the AnaCTDH-based system to take up, transport and deliver carotenoids from lipid membranes into other compartments. We assume that the protein–chromophore interactions that allow AnaCTDH to bind carotenoids lacking keto groups in one (ECN) or both β-ionone rings (like β-carotene) are relatively weak as holo-forms appear only at a large protein excess. We scrutinized the process of carotenoid uptake by AnaCTDH from artificial membranes and showed that ECN distribution is in a dynamic equilibrium, which is shifted from the protein to membrane (35% vs. 65%, respectively), permitting efficient delivery of carotenoids into membranes (Figure 6). Moreover, light could be potentially used in order to activate the process of carotenoid delivery into membranes by AnaCTDH from the photoconvertible OCP [24].

Carotenoids are excellent natural antioxidants, but their delivery to vulnerable cells is challenging due to their hydrophobic nature and susceptibility to photodegradation. Thus, systems securing antioxidant stability and facilitating targeted delivery are of great interest for the design of medical agents [13,15,41,42,43,44,45]. In this work, we have demonstrated that AnaCTDH can deliver ECN into membranes of liposomes and mammalian cells with almost 70% efficiency, which, in Tet21N cells, alleviates the oxidative stress under ROS challenge conditions. Our findings warrant the robustness of the protein-based carotenoid delivery for studies of carotenoid activities and effects on cell models. Alongside with the unseen delivery efficiency, the remarkable stability of OCP-like proteins, the outstanding long-term stability of the carotenoid when embedded in OCP-like proteins, the carotenoprotein’s excellent solubility in aqueous media, and the rapid carotenoid release rates to membranes (minutes) comparing to transfer from liposomes (days [11]), the greatest advantage is the ability to construct genetically encoded modular systems exploiting a toolbox of different functional modules. To exemplify this, we have used the TagRFP-AnaCTDH chimera, where the addition of the bulky TagRFP module (~26 kDa) to the N-terminus of AnaCTDH (~15 kDa) did not break the carotenoid binding and transfer capacity even without construct optimization. 

The use of cyanobacterial water-soluble proteins seems encouraging for numerous biomedical applications and can benefit from their tolerance to lyophilization and astonishingly long shelf-life (from our experience, OCP-related carotenoproteins can sustain years in the fridge). Last but not least, the ability of AnaCTDH to extract CAN from membranes could potentially be utilized for curing pathological conditions like canthaxanthin retinopathy associated with the adverse accumulation of this dietary carotenoid in tissues.

## Appendix A

### ***Spectroscopic Characterization of the AnaCTDH Apoprotein Species***

In contrast to the dimer formed from OCP-CTDs (also called COCP [38]), almost exclusively binding CAN [38,46], AnaCTDH apoprotein may bind both ECN and CAN, which confers different spectral properties and colors to the corresponding holoprotein forms [24]. To monitor the dynamics of holoform assembly and kinetics of the carotenoid transfer, we first studied the spectroscopic signatures of ECN and CAN in different environments in more detail (Figure A1). CAN bound to an AnaCTDH dimer shows the largest red shift of the absorption maximum among all OCP species. The S_0_-S_2_ absorption maximum located at ~560 nm is red-shifted by ~10 nm compared to the corresponding transition of CAN in the non-natural OCP-CTD dimer (COCP) from *Synechocystis* [38,46]. The characteristic ~30-nm difference in the position of S_0_–S_2_ absorption maximum of CAN- and ECN-containing AnaCTDH samples has been reported recently [24]. This indicates that CAN has a higher conjugation length in AnaCTDH, likely due to the presence of keto groups at each of the two β-ionone rings, which both could potentially be conjugated with the polyene chain and involved in hydrogen bonding with Tyr-27 and Trp-110 residues (Tyr-201 and Trp-288 in *Synechocystis* notation), while ECN has only one keto oxygen. We assume that the nature of the large bathochromic shift of CAN absorption in AnaCTDH is due to hydrogen bonding, since mutation of the critical Trp in COCP caused a similar blue shift of CAN absorption (Figure A1A and [46]). Absorption of ECN and CAN in liposome membranes is blue-shifted by ~90 nm compared to AnaCTDH holoproteins, and almost identical to the absorption of these carotenoids in organic solvents [47].

In order to further assess the differences in configurations of ECN and CAN in AnaCTDH and membranes, we used resonance Raman spectroscopy. Although the sequence and secondary structure of COCP and AnaCTDH are very similar, the environment for CAN is not exactly the same, since Raman spectra are different. In addition to the already discussed differences of S_0_-S_2_ absorption, differences in conjugation length were estimated by the position of the ν_1_ Raman band. Given the experimentally observed ν_1_ band positions and the empirical formula ν_1_ = 1459 + 720/(*N* + 1), [48], the number of conjugated double bonds (*N*) of CAN changes from 10.7 in membranes to 13.5 in AnaCTDH, which means that in AnaCTDH, all 13 double bonds, including -C=C- and -C=O in the β-ionone rings, are likely coplanar with the polyene chain. In contrast, the β-ionone rings must be out of conjugation when the carotenoid is embedded in a lipid membrane. Thus, we postulate that the transition of the carotenoid from a membrane into the protein is accompanied by a rotation of the β-ionone rings, which results in an increase of conjugation length and thus leads to a characteristically different absorption spectrum. Since our goal was to study transfer reactions of carotenoids between lipid membranes and proteins, and because there is a simple dependence of the absorption λ_max_ and the shift of the ν_1_ Raman band (Figure A1E), we can use any of these spectral characteristics to infer the state of the carotenoid.

The similarity of the spectroscopic properties of AnaCTDH(ECN) and COCPW288A(CAN) variant [38] indicates that the absence of hydrogen bonds in one of the AnaCTDH subunits (coordinating one of the ECN β-ionone rings, which does not carry a keto group) is equivalent to the absence of two H-bonds with Trps in each of the COCP subunits, resulting in a decrease of the number of conjugated double bonds by~1, comparing to AnaCTDH or COCP with CAN. Such an estimation of the conjugation length changes indicates that even in the absence of hydrogen bonds in one of the AnaCTDH(ECN) subunits the protein affects the conformation of β-ionone ring, which is at least partially in plane with the polyene chain.

### ***Carotenoid Delivery by AnaCTDH does not Require Internalization of the Protein***

Since we observed that the AnaCTDH apoprotein interacts weakly with liposome membranes and that the AnaCTDH holoprotein can deliver ECN into membranes of intracellular organelles, the question arises whether the presence of a carotenoid stabilizes the contacts of the protein with the membrane in addition to anchoring by the CTT. This question is related to the fact that the differences between the environment of ECN in the dimeric AnaCTDH holoprotein and in the membrane are significant (Figure A1), so the transition of carotenoid between these states requires consideration of intermediate protein-carotenoid-membrane complexes. In an attempt to detect such complexes experimentally, we designed a chimeric protein by fusing AnaCTDH and a red-fluorescent protein (TagRFP) according to procedures described in [49]. In such a chimera, TagRFP serves as a fluorescent reporter, the quantum yield of which is sensitive to the presence of the carotenoid in AnaCTDH. Incorporation of ECN or CAN causes a decrease of TagRFP lifetime, which can be used for calculations of the efficiency of excitation energy transfer between the chromophore of TagRFP and the carotenoid. The results of such an analysis even show a different efficiency of FRET in systems with CAN and ECN as chromophores, since consideration of the difference in the absorption of these carotenoids results in similar distances between the chromophore of TagRFP and the carotenoid (~52 Å) in both systems. Although we were able to detect the appearance of the carotenoid in the AnaCTDH part of the chimera, we could not find evidence of colocalization of the chimera at the cell surface or inside the cell with or without carotenoids using fluorescence lifetime imaging microscopy (FLIM). This observation indicates that the lifetime of any protein-carotenoid-membrane intermediate is short (AnaCTDH spends most of the time in solution), which is in agreement with the effects of AnaCTDH interactions with membranes (see Figure 1). Also, the analysis of FLIM data shows that addition of the TagRFP-AnaCTDH chimera to a suspension of cells, which were already enriched by ECN, results in the reduction of the TagRFP fluorescence quantum yield only in a fraction of the protein (~30%), which means that there is a dynamic equilibrium between the delivery and uptake of the carotenoid. Since the presence of the carotenoid in cell membrane does not affect the distribution of TagRFP-AnaCTDH, we suggest that the elementary act of carotenoid translocation from the membrane into the protein occurs rapidly; however, the frequency of such events is low, and, thus, the overall rate of carotenoid delivery is also relatively low (see Figure 3).
